# *“We wouldn’t of made friends if we didn’t come to Football United”:* the impacts of a football program on young people’s peer, prosocial and cross-cultural relationships

**DOI:** 10.1186/1471-2458-13-399

**Published:** 2013-04-27

**Authors:** Sally Nathan, Lynn Kemp, Anne Bunde-Birouste, Julie MacKenzie, Clifton Evers, Tun Aung Shwe

**Affiliations:** 1 School of Public Health and Community Medicine, UNSW, Sydney, NSW, 2052, Australia; 2 Centre for Primary Heath Care and Equity, UNSW Sydney, NSW, 2052, Australia; 3 International Communications Department, University of Nottingham, Ningbo, China

**Keywords:** Sport-for-development, Community-based intervention, Impact evaluation, Peer relationships, Prosocial behaviour, Cross-cultural relationships

## Abstract

**Background:**

Sport as a mechanism to build relationships across cultural boundaries and to build positive interactions among young people has often been promoted in the literature. However, robust evaluation of sport-for-development program impacts is limited. This study reports on an impact evaluation of a sport-for-development program in Australia, Football United®.

**Methods:**

A quasi-experimental mixed methods design was employed using treatment partitioning (different groups compared had different levels of exposure to Football United). A survey was undertaken with 142 young people (average age of 14.7 years with 22.5% of the sample comprising girls) in four Australian schools. These schools included two Football United and two Comparison schools where Football United was not operating. The survey instrument was composed of previously validated measures, including emotional symptoms, peer problems and relationships, prosocial behaviour, other-group orientation, feelings of social inclusion and belonging and resilience. Face to face interviews were undertaken with a purposeful sample (n = 79) of those who completed the survey. The participants in the interviews were selected to provide a diversity of age, gender and cultural backgrounds.

**Results:**

Young people who participated in Football United showed significantly higher levels of other-group orientation than a Comparison Group (who did not participate in the program). The Football United boys had significantly lower scores on the peer problem scale and significantly higher scores on the prosocial scale than boys in the Comparison Group. Treatment partitioning analyses showed positive, linear associations between other-group orientation and total participation in the Football United program. A lower score on peer problems and higher scores on prosocial behaviour in the survey were associated with regularity of attendance at Football United. These quantitative results are supported by qualitative data analysed from interviews.

**Conclusions:**

The study provides evidence of the effects of Football United on key domains of peer and prosocial relationships for boys and other-group orientation for young people in the program sites studied. The effects on girls, and the impacts of the program on the broader school environment and at the community level, require further investigation.

## Background

Australia accepts approximately 14,000 people either as refugees or on humanitarian grounds each year [1]. Of those who are accepted into Australia’s Refugee and Humanitarian program, just under one third settle in New South Wales [2] - Australia’s most populous state. Western Sydney is one of Australia’s most culturally diverse areas, with almost 40% of the population born in other countries [3]. A large proportion of those who are accepted into Australia as refugees or humanitarian entrants are aged 17 or below (35% of offshore visas granted in 2010–2011 were to people aged 0–17) [4]. Young people’s experience of settlement in Australia, including entry into the school educational system, is significantly distinct to that of adult arrivals [5,6]. As with adults, young people may need to learn a new language and negotiate a different culture, but also face the additional challenges of their development stage, educational pressures and the centrality of peer relationships to their experiences [5,6]. How and to what extent peer and social bonds are formed amongst young people who are newly arrived in Australia, and between these young people and other young people in their school or community, represents an important area of study.

Previous studies have contended that sport may assist to build relationships across religious, ethnic and economic lines [7], as well as act as a mechanism to strengthen community, reduce crime rates, and provide mentoring and support for individuals [8-11]. However, at the time of this study’s inception there were few, if any, robust studies which assessed sport-for-development program impacts on young people, including those with refugee experiences, highlighting the need to evaluate sport-based programs to assess their effects. [7,8,12,13]. For the purpose of this paper we are defining sport–for-development as the use of sporting activities to provide opportunities for personal and community development with aims that go beyond the sphere of physical activity and [elite] player or game development.

Coalter’s review of sport-for-development programs [8] found that reported impacts on health, crime, employment and regeneration were largely anecdotal, and reliance on anecdotal evidence has continued to be noted in more recent reviews in Australia and overseas [7,14,15]. Research published in 2010 and 2011 continues to highlight the lack of rigorous measurement and evaluation of sport-for-development programs [15-17]. In addition, designing evaluation approaches that can help researchers and practitioners understand the complex and interactive effects of sport-for-development programs continues to be a challenge [16]. Levermore notes that the number of evaluation studies has increased [16]. However, both Coalter and Levermore warn that evaluation in this field is fraught – most studies are process orientated, looking at outputs not impact, and insecure funding environments creates pressure to demonstrate positive ‘outcomes’ [16,17].

This article reports on the impacts reported by young people who participated in a school-based sport–for-development program, Football United, compared to those who did not participate, but attended schools in a similar setting [18]. The study was designed to directly address past criticisms of program evaluation [18] with the findings reported here drawn from a mixed-methods quasi-experimental design.

### Program description: Football United®

The Football United® program is a complex, multi-level football intervention targeted at young people in culturally diverse areas such as the western Sydney region with high levels of refugee settlement. The choice of football as the vehicle in this program was purposeful, as it is relatively inexpensive, is played in many countries, including those of many recently arrived young people with refugee experiences, and by both genders.

The Football United program utilises a social-ecological framework, working with an awareness that heath and social behaviour is affected by, and in turn affects, the formal and informal social, cultural, physical and institutional relationships in which they are located [19-21]. Football United aims to intervene at these multiple levels, and to do so operates in dialogue and partnership with schools, migrant and refugee support organisations, football organisations, community groups, corporations, and most importantly with young people themselves.

Football United currently operates in a number of sites in Australia. There has been significant growth in the program’s reach and depth in recent years – it began in 2006 with one community park-based site and now has 13 program sites in New South Wales, with further sites in three other states in both urban and rural areas. The Football United sites evaluated in this study were based in Intensive English Centres (IECs) and Host High Schools where IECs are located in Western Sydney. IECs are part of the state school system and prepare newly arrived secondary aged students for study in an Australian high school by providing intensive English tuition. There are 13 IECs in Sydney, each attended by students from a broad geographic catchment area who are referred by their local high school. Students can spend up to five terms in an IEC before transitioning to their local high school.

Students involved in Football United can play football after school at these sites, as well as access additional program components including training as football coaches, life skills and leadership development workshops. The program aims to promote participants’ health and well-being, social inclusion, connectedness, and cross-cultural engagement.

The Football United program has four key focus areas:

1. Football activities: Regular Saturday and after school training, school holiday camps, competitions and festivals are a central program activity. Mentorship between coaches, volunteers and players is actively promoted in all activities.

2. Capacity building: Members of local communities participate in free training in coaching and refereeing, mentoring and life-skills, leadership and project management and apply their learning in the program. A significant proportion of young people who participate as players in the program continue with the program as volunteer or paid coaches and project coordinators.

3. Building linkages: Linkages between program participants and partner agencies, including local football clubs, government, community and corporate sectors are a focus of the program.

4. Creating awareness of Football United and issues for communities: This is achieved through advocacy, key partnerships and individual high profile champions.

Football United is coordinated by an overarching management team, with paid and volunteer coaches delivering the program at each of its sites and liaising with the community. Student participants who have completed coaching courses are eligible to coach junior participants on a voluntary basis. One or more school staff members act as coordinators and liaisons between the different sites, and the program management team. More details about the program can be found at http://www.footballunited.org.au/

## Methods

This research study utilised a quasi-experimental mixed methods design and the methodological approach aligns with the socio-ecological framework underlying the Football United program. To effectively evaluate this program we needed to use a variety of measures at different levels of impact [18] and actively collaborate with the multiple organisations and partners involved in its design and delivery, including school and migrant resource centre staff. Consultation included participation in two key workshops conducted in the study design phase. As the study progressed, a Research Reference Group comprising representatives from grant partners and participating schools formally met annually to advise on proposed study measures and recruitment of study participants. The research team included a mix of those engaged in the design and management of the program as well as researchers independent of the program. The researchers engaged in field work and primary analyses were not involved in program management and delivery at the time of the study.

The study was approved by the University of New South Wales Human Research Ethics Committee and by the State Education Research Approvals Process. This paper reports only on the data collected from young people. Relevant methods and measures are described below and details about the full study measures and approach have been published previously [18].

### Aims and hypotheses

The Football United study’s broad goal was to investigate the implementation of the program and to analyse processes and impacts on individual health and well-being, social inclusion and cohesion. The overall study had four aims and two hypotheses [18]. The single aim and hypothesis addressed in this paper are:

#### Aim

To determine the impact of Football United on participants’ personal development, emotional health, resilience, social inclusion and peer relationships.

#### Hypothesis

Participants in the Football United Program will have significantly better emotional health, peer relationships and feelings of social inclusion than those who do not participate at all or who only participate minimally in the program.

### Study design

The study was a quasi-experimental design with treatment partitioning, which means the different groups compared in the study had a different level of exposure and involvement in the Football United program. This design was important to address the particular challenges of working with a study population that is always changing, with new Football United participants arriving regularly and moving locations, and with a program that had already been in operation for three years. These factors meant that a true baseline measurement was not possible and a study design with treatment partitioning represented the best approach to enable causal inference and protect internal validity [22]. The approach compared those with no involvement in the Football United program - called the Comparison Group - with those with different levels of participation in Football United activities, referred to as the FUn Group. The Comparison group was measured at a single point in time enabling them to participate in the program following measurement. This was important to meet ethical requirements of reciprocity [23].

The FUn Group comprised students from two IECs and their host high schools in Sydney, Australia who had participated in the Football United program prior to and during the study. The Comparison Group comprised recent arrivals to Australia who had attended an IEC or its host high school at two sites in demographically similar areas, and where Football United was not operating at the time of the study.

### Sample selection and recruitment

All participants at the two Football United schools and all students at the two Comparison schools who had arrived in Australia as refugee or humanitarian entrants were eligible to participate in the overall research study. At Football United sites, the study was introduced to participants directly by IEC staff. At Comparison sites the study was introduced through staff and during school assemblies Particular attention was paid to align demographics as closely as possible between the two groups. For example, at one Football United site study participants included former IEC students who had entered Australia as refugee entrants who had transitioned to the host high school. The sample for the Comparison site was thus recruited accordingly to include some students who were now attending the host high school.

The study population is vulnerable and some may have experienced trauma or persecution prior to arrival in Australia, sometimes in the form of governmental abuse. To be sensitive to this vulnerability, the research proceeded with an iterative model of consent that treated the process of ensuring consent as more complex than simply signing a form [24]. This approach aligned with the underlying program philosophy of building capacity in participating communities and enabling them to learn about research and the consent process. Participation in the study required the consent of the young people involved, as well as their parent or guardian. Bi- and multi-lingual school support staff were actively engaged in the process of gaining informed consent.

### Measures

The complexity of the Football United program posed challenges for measurement and evaluation. Recognition that program impact is often not straightforward or linear [25] meant that too heavy an emphasis on measuring specific pre-determined outcomes would not enable understanding of the range of possible effects of the program. Measuring change required close attention to multiple levels [26-28] drawing on mixed methods and multidisciplinary approaches [29,30]. The program complexity and plasticity was mirrored by a flexible and emergent study design [31], in particular in participant recruitment and methods employed. Plasticity refers to “the capacity of programs and peoples to alter their action and experiences in response to changes in their environment and context. This property can be studied at the micro- and macro-level of program implementation” (p 2) [18]. Different measures were employed to provide data across a range of levels of possible impact of the program [18]. The measures chosen for the survey were based on areas of greatest potential impact of the program agreed among the research team together with program partners, including staff at schools and migrant resource centres. These hypothesised impact areas were chosen as they matched core program objectives, for example; improving the relationships between young people as peers, including among those from diverse cultural backgrounds; increasing mentoring, volunteering and contribution to others through playing and capacity building activities. Measures related to the participant data reported in this paper are detailed below.

### Designing and adapting the survey tool

A survey instrument comprising a composite of established measures was developed for the study [18]. It measured emotional well being, specifically strengths and difficulties [32] which includes scales on peer problems or relations (included questions about being bullied and friendships with peers), prosocial behaviour (included questions about sharing with others, caring about others feelings, being helpful if someone is hurt or upset, and volunteering to help others), hyperactivity/inattention and resilience on a 3 point likert scale (not true to certainly true) [33,34]. A scale on conduct problems, such as lying and cheating, which was part of the Strengths and Difficulties Questionnaire was not included on advice from partner schools that it would be confronting and upsetting for the students. The survey also included measures of ethnic identity/other-group orientation [35-37] and feelings of social inclusion and belonging [38,39]. The other-group orientation scale asked about whether participants like meeting and getting to know people from ethnic groups other than their own and being around and spending time with them using a five point likert scale (strongly agree to strongly disagree). The survey instrument was developed in close consultation with partners including attention to face validity, match of construct measured to hypothesised program impacts and psychometric properties as detailed in Additional file 1: Instruments and items chosen for survey and rationale for choosing, and as discussed in a previously published paper [18]. The survey was made appealing and accessible by including two important elements; practice questions and engaging visuals, and a pilot was undertaken with a group of IEC students. Demographic data modelled on the questions used in the recent Good Starts Study in Victoria [40] was also collected including age, country of birth, language spoken, date of arrival, country prior to arrival, and level of English spoken prior to arrival. The English survey format, including key measures where reproducible under copyright law, can be found at http://www.footballunited.org.au/research/arc/research-questionnaire. The full survey was translated (and back-translated to assess accuracy) into Fasi and Dari, two key language groups represented among the study population. All three language versions were pilot tested with a small group of young people at Football United schools and found to be suitable for use with young people in IEC schools with little difficulty reported in completing the survey.

### Quantitative data collection

The survey was administered with bilingual support as needed with the type of assistance recorded. After the first round of data collection with the FUn Group, the ethnic identity scale in the Multigroup Ethnic Identity Measure was removed from the survey [35]. With wider use in the study, the scale was found to create confusion amongst some participants who had difficulty identifying their ethnic identity, yet had little difficulty in thinking about people from ‘ethnic groups other than their own’. The other-group orientation scale in this measure was therefore retained. The final instruments and items used are summarised in Additional file 1 with additional details to support selection and use of these measures in the present study. The survey instrument was administered to all consenting participants.

### Qualitative data collected

A sub-sample of those who completed the survey instrument at all four schools also participated in an interview, often with one or more of their peers [18]. The participants in the interviews were purposefully selected from the larger survey sample to provide a cross-section of age, gender and cultural background. All of those approached to participate in the interviews were able to be interviewed once a suitable time was arranged. Interviews were audio-taped with participants’ consent and conducted in English, but where interviewees’ English communications skills were limited, bilingual school support workers acted as interpreters. The interview responses were recorded and transcribed in English, not other languages. Only some of the interviewees required support at a few points with communicating their experiences. When this occurred, interpretation into English from the language spoken by the participant, was undertaken during the interview by bi-lingual support staff with good rapport and trust with participants. Checking back with respondents that their experiences and views were captured adequately was also undertaken by the bi-lingual support staff following their interpretation to English. These staff were instructed to translate the questions as closely as possible and give as close to verbatim translation of responses into English. While translation inevitably meant that participants’ views were mediated, the team took steps to ensure young people’s perspectives were understood as best as possible, also using drawings, brainstorming exercises and role plays to capture their views. The interviews were informal and unstructured with interviewees asked open-ended questions ranging from their previous countries of residence and length of time in Australia, their families and neighbourhoods, their experiences meeting and making friends, as well as their experiences with and feelings about sport in general and the Football United program specifically (for FUn Group only). The interviews covered each of the topic areas listed though often in a different order and manner depending on how the interview proceeded, an approach which is typical of qualitative research with a focus on responsiveness to the individual and each unique experience [31]. The research team was alert to the potential impact of social desirability - that is, participants wanting to appear positive about the Football United program, their school or Australia as a country [31]. To address this, the consent process involved informing participants that their responses would be de-identified, and that what they said would not affect their future participation in Football United. This was also emphasised during data collection. In addition, questions were framed to allow participants to suggest how the program could be improved, rather than directly criticise its operation. Participants were also asked about what ‘others they knew’ thought about the program to allow them to create distance between comments made and themselves [31].

#### Data analysis

The study was enriched and strengthened by the use of two data sources: a survey and interviews as described above, and three different analyses: by group membership, level of participation, and qualitative analysis, each of which is described below. The findings of each analysis is presented in the results and then drawn together in the discussion. As suggested by the metaphor of triangulation, the confirmation of findings by two or more independent measurement processes greatly enhances confidence in the research findings [41].

### Survey analysis

Descriptive quantitative analysis of the demographic characteristics of the FUn and Comparison Groups and univariate analysis of impact measures were undertaken. Two analyses were then undertaken to assess the impact of the Football United program. Firstly, an intention to treat analysis was undertaken to compare the outcome measures between young people enrolled at the participating intervention schools (regardless of whether they attended program activities or not) and those who completed the survey at the Comparison schools. Secondly, a treatment partitioning analysis was undertaken to explore the impact of no, lower or higher participation in Football United activities on the outcome measures. Analysis of Variance (ANOVA) was used for scale outcome measures and Chi-square tests for proportional outcome measures. In addition, factorial ANOVA was used to determine interactive effects of demographic variables on measured impacts.

### Interview analysis

Qualitative interview data were transcribed in full with a unique identifier attached to each participant to allow comparison between survey and interview data. For each quote a brief descriptor is provided about the participant including whether they were from a Football United School (FS) or Comparison School (CS) and their gender. The quotes are taken from young people from a diverse range of countries, but country of birth has not been provided for each quote to protect anonymity. The overall qualitative data set was first analysed inductively and thematically with constant comparison undertaken, that is, comparison of the views of interview participants within and between schools [31]. NVIVO 9, a qualitative data management tool, was used by the main field researcher [42] and all data coded to nodes created iteratively with movement between the data set and nodes. Discussion between the field researcher and lead investigator was ongoing throughout the initial coding and this assisted in the refinement of nodes and identification of key themes. Analysis for this paper focussed specifically on whether the qualitative data set provided support or not for the quantitative findings including searching for ‘negative cases’[31]. Data which referred to friends, peer relationships in general (both positive and negative accounts), prosocial behaviour, other group orientation and resilience were also examined in detail using the constant comparison technique. The two main researchers shared this data set with the rest of the research team and discussions about the qualitative findings were a focus of three team research meetings which guided subsequent refinement of the analysis for this paper. Matrix queries in NVIVO 9 were also used to look at the attributes (school type and gender in particular) of young people who made different types of comments that related to the survey domains measured, such as peer relationships, prosocial and other group-orientation, which were created as nodes during later stages of the analysis.

## Results

### Survey sample

Response rates to the survey for Football United schools’ were 76% (site 1) and 54% (site 2) of all Football United participants in the survey year. At the Comparison schools the response rate was less important (overall around 25% of IEC students sampled) as the sample was recruited to try to minimise difference in demographic characteristics between this sample and the FUn group, prioritising comparability over representativeness. The Comparison school sample frame was much larger than that at Football United schools as it included the whole IEC student population, versus a sub-set of IEC students at the Program schools who participated in Football United as the sampling frame. The larger sampling frame for the Comparison schools participants is therefore reflected in a lower calculated response rate. Participant demographic characteristics and immigrant experience are outlined in Table 1. The average age of participants across all sites was 14.7 years (SD = 2.4). Only three girls participated in the survey at the first Football United school site, due to only a small number of girls participating in Football United that year at this site. As recruitment in the intervention group was limited to those participating in Football United at these schools, the research team agreed to focus efforts on recruiting boys to complete the survey. Results are reported for both the full sample and for boys only. Participants from both FUn and Comparison Groups had lived previously in countries including Iraq, Iran, Afghanistan, Sierra Leone and other African nations, Pakistan, Burma and Thailand. The Comparison group were significantly more likely to self report as refugees or asylum seekers and to have lived in two or more countries before arriving in Australia. There were no significant differences in participants’ schooling or language knowledge prior to arrival in Australia.

**Table 1:** Demographic, immigrant experience and current social environment characteristics

**Demographic characteristics**	**FUn**	**Comparison**	**Statistic**	**P**
Age mean (SD)	15.0 (2.6)	14.4 (2.1)	t_2,133_ = 1.49	0.14
Gender n (%)			χ^2^_1_ = 20.49	<0.001
Male	60 (95.2)	50 (63.3)		
Female	3 (4.8)	29 (36.7)		
Country of birth n (%)				
Afghanistan	4 (6.6)	29 (37.2)		
Burma	1 (1.6)	9 (11.5)		
Iran	5 (8.2)	5 (6.4)		
Iraq	23 (37.7)	3 (3.8)		
Sierra Leone	3 (4.9)	17 (21.8)		
other African	11 (18.0)	7 (9.0)		
other Asian	8 (13.1)	7 (9.0)		
other	6 (9.8)	1 (1.3)		
**Immigrant experience**				
Lived in 2 or more countries before coming to Australia			χ^2^_1_ = 4.64	0.03
One country	28 (47.5)	23 (29.5)		
2 or more countries	31 (52.5)	55 (70.5)		
Year of arrival in Australia			χ^2^_2_ = 25.98	<0.001
this year	17 (27.4)	25 (31.6)		
last year	21 (33.9)	50 (63.3)		
more than one year ago	24 (38.7)	4 (5.1)		
Refugee or asylum seeker			χ^2^_1_ = 7.66	0.006
not refugee or asylum seeker	15 (25.9)	6 (8.1)		
refugee or asylum seeker	43 (74.1)	68 (91.9)		
Years of schooling before arriving in Australia mean (SD)	6.2 (4.1)	6.0 (3.4)	t_2,119_ = 0.25	0.80
Knowledge of English before coming to Australia			χ^2^_2_ = 2.68	0.26
No English	24 (38.1)	20 (25.6)		
A little/some English	31 (49.2)	48 (61.5)		
Very good English	8 (12.7)	10 (12.8)		

### Interview sample

Interviews were conducted with a sub-sample of participants in both FUn and Comparison survey groups. Interview participants ranged in age from 11–18, and had arrived in Australia from a diverse array of countries including Iraq, Iran, Afghanistan, Pakistan, Sierra Leone, Liberia, Sudan, Eritrea, Nepal, Burma and Thailand. The sampling focussed on selecting a diversity of students including girls. The FUn Group interview sample included 48 young people from 20 different countries of birth and included nine girls. The Comparison Group sample included 31 young people from 12 different countries of birth and included nine girls.

### Participation in and experience of Football United

Thirteen (13) young people from the Football United schools were recorded as not participating in any Football United activities (that is, they enrolled, but did not attend any weekly after school activities) during the study year. Fifty (50) young people participated in at least one Football United activity during the study year (79.4%). On average, participating young people attended just over sixty percent (61.7%) of activities in each 10 week term for which they were registered as a Football United participant - regularity of attendance (Table 2). During the data collection period of three school terms (when the program operates), each participating young person’s total attendance was, on average, the equivalent of full attendance for over one and half school terms - total participation (1.6). There were no significant differences in the demographic characteristics or immigrant experiences of those participating young people with none, lower or higher average attendance or no, lower or higher total participation, with the exception that those young people who had arrived in Australia more than one year ago were, not unexpectedly, more likely to have higher total participation (χ^2^_4_ = 9.5 p = 0.05). Only 13 young people in the survey sample completed the survey in Dari or Fasi with the remainder completing the survey in English. Most of the participants required little or no support with completing the survey, although bi-lingual support staff assisted 13 participants at the Program School and 18 at the Comparison School in completing the survey tool.

**Table 2:** Participation in and experience of Football United (FUn group only)

**Participation**	**n**	**%**
Did not participate in previous year	13	20.6
Participated in FUn activities in previous year	50	79.4
**Levels of participation for participating young people (n = 50)**	**mean (sd)**	median*
Regularity of attendance per term†	0.62 (0.26)	0.66
Total participation over previous year^‡^	1.59 (0.90)	1.46
**Experience**	**n**	**%**
Feel better since coming to Football United		
Same or worse	10	14.8
A bit or much better	52	83.9
Football United help in other ways		
Not at all or only a little	9	14.8
Quite a lot or a great deal	52	85.2

† calculated as the average (mean) proportion of activities in which the young people participated for each term in which they were registered as a FUn participant: potential range from any number greater than zero to one (1), where one (1) means the young people participated in every activity in the school term/s in which they were registered as a FUn participant.

‡ calculated as the total sum of the proportion of activities in which the young people participated for the duration of their participation in the study year: potential range from any number greater than zero to four (4), where four (4) means the young people participated in every activity in all four school terms.

* Fifty (50) percentile cut point for determining lower and higher participation/attendance.

The young people in the FUn group reported feeling better since commencing in the program (Table 2), and reported that the program had helped them. Feeling better since coming to Football United was significantly positively correlated with both regularity of attendance and total participation in the program (r^2^ = 0.31 p = 0.02; r^2^ = 0.32 p = 0.01 respectively). In interviews, participants in the FUn Group did not directly mention ‘feeling better’ since coming to Football United, but many talked about *“having fun”* (FS, Male) and *“making friends”* (FS, Male). The following quotes typify the kinds of comments made in interviews:

When I play soccer, I’m feeling like very nice, so nice. I can’t explain. Just that feeling’s so different. When I play soccer, I’m feeling so happy (FS, Male).

Yeah, it helped me because I wasn’t know very much about football. I just go and they teach me how to play, and I got lots of fun for friends and playing, and jumping around, it’s so good (FS, Male)

And it’s fun like meeting new friends. People who we haven’t met before. (FS, Girl)

### Impact by schools attended

The impact for all study participants, comparing young people from the FUn Group and Comparison Group, are outlined in Table 3.

**Table 3:** Outcomes for all participants

**Outcome**	**FUn**	**Comparison**	**Statistic**	**P**	**Effect size**	**Power**
Strengths and difficulties, mean (SD)						
Emotional symptoms^†^	3.27 (2.61)	3.32 (2.07)	t_2,133_ = 0.13	0.90	0.02	0.06
Hyperactivity^†^	2.88 (2.09)	2.96 (1.66)	t_2,129_ = 0.24	0.82	0.04	0.08
Peer problems^†^	2.95 (2.05)	3.19 (1.57)	t_2,131_ = 0.75	0.46	0.13	0.18
Prosocial behaviour^‡^	8.56 (1.44)	8.05 (2.21)	t_2,136_ = 1.64	0.10	0.27	0.50
Other-group orientation^†^, mean (SD)	1.80 (0.62)	2.12 (0.68)	t_2,140_ = 2.30	0.006	0.46	0.90
Resilience^‡^*, mean (SD)	3.59 (1.07)	3.69 (1.06)	t_2,138_ = 0.54	0.59	0.09	0.14
Social inclusion, mean (SD)						
Most people can be trusted†	2.48 (0.92)	2.29 (0.95)	t_2,138_ = 1.15	0.25	0.20	0.33
Run into friends in the local area†	2.48 (0.96)	2.56 (0.84)	t_2,138_ = 0.53	0.59	0.09	0.13
Social inclusion, n(%)						
Have family members living in the neighbourhood			χ^2^_1_ = 3.26	0.07	0.01	
Yes	34 (54.8)	55 (69.6)				
No	28 (45.2)	24 (30.4)				
Have close friends in the neighbourhood			χ^2^_1_ = 0.07	0.79	0.01	
Yes	32 (51.6)	39 (49.4)				
No	30 (48.4)	40 (50.6)				
Know people living in the neighbourhood			χ^2^_1_ = 2.56	0.11	0.01	
Many or most	41 (66.1)	58 (78.4)				
Few or none	21 (33.9)	16 (21.6)				

^†^ Lower score is more positive response, ie.fewer emotional symptoms, less hyperactive behaviour and fewer peer problems.

^‡^ Higher score is more positive response, ie. more prosocial behaviour and more resilient.

* Ability to adapt to changes and bounce back after illness, injury or other hardships.

Overall, participating young people from the FUn Group reported being significantly more other-group orientated than the Comparison Group. There were no significant differences between the groups in participants’ reporting on any other survey measures. Factorial ANOVA was used to determine interactive effects of demographic and immigrant experience variables on measured impacts, for those where there were significant differences between the two study groups in gender and immigrant experience. Gender significantly impacted only on reporting of emotional symptoms overall, with girls reporting a higher score than boys (girls mean 4.23 (SD 2.2), boys mean 3.0 (SD2.3), t_2,133_ = 2.92 p = 0.004). This did not impact on group differences between the FUn and Comparison Group, however, due to the small number of girls in the FUn group, analysis of all impact was also conducted for boys only (see Table 4). For boys, the FUn Group reported significantly more positive responses than the Comparison Group, with medium to large intervention effects for peer problems, prosocial behaviour and other-group orientation.

**Table 4:** Impacts for male participants

**Impact**	**FUn**	**Comparison**	**Statistic**	**P**	**Effect size**	**Power**
Strengths and difficulties, mean (SD)						
Emotional symptoms^†^	3.08 (2.47)	2.85 (2.01)	t_2,101_ = 0.53	0.60	0.11	0.13
Hyperactivity^†^	2.80 (2.12)	2.96 (1.60)	t_2,100_ = 0.40	0.69	0.08	0.11
Peer problems^†^	2.84 (1.93)	3.58 (1.67)	t_2,100_ = 2.02	0.046	0.40	0.67
Prosocial behaviour^‡^	8.63 (1.42)	7.80 (2.23)	t_2,106_ = 2.40	0.024	0.45	0.73
Other-group orientation^†^, mean (SD)	1.78 (0.58)	2.23 (0.69)	t_2,108_ = 3.72	<0.001	0.67	0.98
Resilience^‡^*, mean (SD)	3.08 (2.47)	2.85 (2.01)	t_2,101_ = 0.53	0.60	0.11	0.13
Social inclusion, mean (SD)						
Most people can be trusted†	2.44 (0.91)	2.36 (1.06)	t_2,107_ = 0.43	0.67	0.08	0.11
Run into friends in the local area†	2.46 (0.95)	2.56 (0.81)	t_2,107_ = 0.60	0.55	0.11	0.09
Social inclusion, n(%)						
Have family members living in the neighbourhood			χ^2^_1_ = 2.60	0.11	0.02	
Yes	33 (55.0)	35 (70.0)				
No	27 (45.0)	15 (30.0)				
Have close friends in the neighbourhood			χ^2^_1_ = 0.15	0.70	0.003	
Yes	31 (51.7)	24 (48.0)				
No	29 (48.3)	26 (52.0)				
Know people living in the neighbourhood			χ^2^_1_ = 2.84	0.09	0.02	
Many or most	39 (65.0)	36(80.0)				
Few or none	21 (35.0)	9 (20.0)				

^†^ Lower score is more positive response, ie.fewer emotional symptoms, less hyperactive behaviour and fewer peer problems.

^‡^ Higher score is more positive response, ie. more prosocial behaviour and more resilient.

* Ability to adapt to changes and bounce back after illness, injury or other hardships.

Outcomes were not significantly related to the number of countries the young people had lived in before Australia, the year they arrived in Australia, or having family, friends or people they know in the neighbourhood, either overall, for males participants only, nor when assessed for interaction effects with group membership, that is by FUn or Comparison Group. Reporting of emotional symptoms was related to refugee/asylum seeker experience, with those reporting refugee/asylum seeker experience reporting a higher score for emotional symptoms than those who did not (refugee/asylum seeker experience mean 3.6 (SD 2.4), no refugee/asylum seeker experience mean 1.8 (SD 1.5), t_2,123_ = 4.49 p < 0.001). This difference did not impact on differences between FUn and Comparison Group participants in impacts either overall or for male participants only.

### Impacts by levels of participation and attendance

Analysis was conducted using two forms of treatment partitioning to assess whether impacts were related to the total amount of participation in Football United or regularity of participation in Football United activities (or both). The three treatment partitioning categories of total participation were:

a. No participation (n = 92), includes Comparison Group (n = 79) and FUn Group non-attenders (n = 13).

b. Lower total participation (n = 25); total participation less than what would be received by a young person fully participating for 1.46 terms (14.6 weeks).

c. Higher total participation (n = 25); total participation equal to or more than what would be received by a young person fully participating for 1.46 terms.

The three categories of regularity of attendance were:

a. No attendance (n = 92), as above.

b. Less regular attendance (n = 25): average attendance in less than two thirds (66 percent) of the sessions per term for the terms in which the young person was registered in the Football United program. Each session is 2 hours meaning there are 20 hours of after-school playing activities offered per term.

c. More regular attendance (n = 25): average attendance in equal to or more than two thirds of the sessions per term for the terms in which the young person was registered in the Football United program.

**Figure 1:**
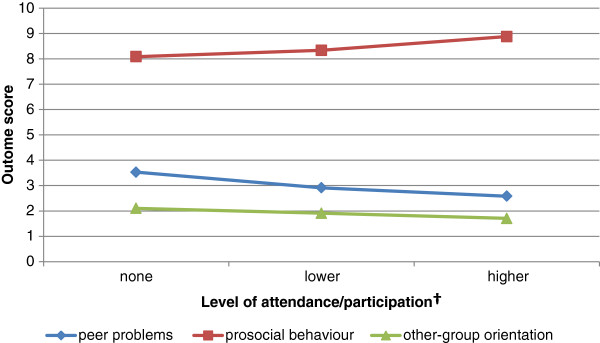
Treatment partitioning correlations - male participants.

For all study participants, other-group orientation was significantly correlated with the level of total participation in the Football United program, with higher total participation associated with lower (more positive) other-group orientation (r^2^ = -0.18 p = 0.03). As above, analyses were also conducted for boys only. For boys, higher total participation had a significant linear association with lower (more positive) other-group orientation (r^2^ = -0.21 p = 0.03). More regular attendance for boys also had a significant linear association with lower scores (more positive) on peer problems and higher scores (more positive) on prosocial behaviour (r^2^ = -0.22 p = 0.03, r^2^ = 0.19 p = 0.05 respectively; Figure 1).

### Interviews

The qualitative data from interviews with participating young people in the FUn and Comparison Groups provides support for the quantitative findings as outlined below. Level of attendance at Football United activities of the interviewee is provided where relevant.

#### Other-group orientation

Many young people in the FUn Group talked about enjoying meeting and engaging with people from other backgrounds, with Football United often explicitly mentioned as a place for meeting a diversity of students:

So when I came here and I started playing Football United, I met people from other countries, Iraq, Congo, Cambodia, and if it wasn’t for soccer … I wouldn’t know these people. So that’s a good thing about Football United (FS, Male).

Yeah and it’s really fun, get to know people from other backgrounds, learn from them (FS, Male).

I like to mix with different people. To know other things, what they do in their country and what they eat and stuff (FS, Girl).

Matrix queries in NVIVO found FUn Group interviewees, with lower and higher regularity of attendance, often talked about the value and benefit of mixing across cultures further supporting the findings around other-group orientation. Comparison Group and FUn group members interviewed who had not participated in any Football United activities didn’t talk much about other-group orientation, rather a number of them commented about being more comfortable with their own culture and language groups, for example:

It’s a little bit hard because of the language, you know, we are not so good in speaking English like them so sometimes we find it difficult to make friends with them … so for now most of my friends from the same country (CS, Male).

The FUn Group with no participation and some interviewees in the Comparison Group sometimes expressed a generalised sense of appreciation of a multicultural society:

To me, the people, and you need to respect each other, countries, and where you're from, no matter where, who you are. You need to respect each other’s cultures and take that responsibility on yourself. Ensure you're not trying to change other people. Yeah (CS, Male).

Some FUn Group interviewees also suggested that participation in Football United had prompted them to respect others as individuals, irrespective of their backgrounds. For example:

Like we learn to respect other people no matter who they are (FS, Male).

And it doesn’t matter who is it, where the player’s from you just play and have fun with him (FS, Male).

#### Peer relationships and problems

Many interviewees in the FUn Group frequently referred to attendance at Football United as a means for them to find and make new friends and overcome peer problems, even those who didn’t attend regularly, as the following quotes demonstrate:

We wouldn’t of made friends if we didn’t come to Football United (FS, Male, low attender).

Yeah, before class they’d pick on us and stuff. But now from Football United we all come together (FS, Male, high regular attender).

*I think it was fun because when I first came here and I didn’t have friends and when I started soccer and that I get friends and more friends from high school* (*FS, Male, lower attender).*

And it’s fun like meeting new friends. People who we haven’t met before. (FS, Female).

The matrix query revealed a gradient in proactivity in peer relationships associated with regularity of attendance – those with low attendance in Football United speak of positive peer relationships, but do not report being as proactive in seeking and engaging in peer relationships as those with high regularity of attendance. The matrix query also revealed that peer problems were not very evident in FUn Group interviews – even amongst those with no participation providing some evidence that Football United’s presence at the school may have an impact on peer relationships at the school reflected in reduced reporting of peer problems.

Comparison Group interviewees often talked about school as the main place that they had met friends. Some had made friends through playing sport at local parks as well as through attending church:

*I: Where do you meet friends? R: At school (CS, Male).I: Anywhere else?R: Probably just the school, and sometimes the park when we saw a lot of friend, because they want to play soccer and then come and just introduce us then we some friend there (CS, Male).*

There were a number of instances of Comparison Group interviewees talking about difficulties connecting with friends after school, as illustrated in the following excerpt:

*R: No, because I don’t go out of the home very much (CS, Male).I: Right. So you mostly just go from school to home?R: Yeah.*

A number of interviewees in both the FUn and Comparison Groups talked about bullying as a problem - their association with the IEC school and lack of proficiency with English were mentioned as catalysts for bullying as the quotes following show:

Like everywhere is bullying, but here, people at high school always are fighting with us, and we don’t like to fight, so we are trying to find a high school where we are safe (FS, Male, non attender).

Because they are like bullying, because we like in the IEC they think they can just bully us (CS, Male).

Even though bullying appears to remain a problem in all schools studied, interviewees in one FUn Group interview observed that Football United had helped to improve the situation:

They say when Football United haven’t start [school named] there’s always a fight between IEC students and the high school students but then when the Football United program started everyone started playing and started talking and try and make a friend, which is good stuff (FS, Male, lower attender).

#### Prosocial behaviour

In the interviews, a number of Football United participants reported enjoying helping and teaching others, especially those who had been in coaching roles, as illustrated in the first two quotes below. The value of student coaches from the participant perspective was also highlighted by some interviewees (last quote):

R: It’s fun and when we learn good we can teach other people (FS, Male).

I’d go coach the little boys. I really enjoyed that. It was nice, yeah. I had fun, too. I was laughing and – yeah, I think it was good that day (FS, Male).

Student coaches, they learn more by like teaching other people [and] they know how it feels (FS, Female).

An NVIVO matrix query revealed differences in prosocial behaviour between lower and more regular attenders of Football United with more regular attenders expressing prosocial behaviour in terms of leadership whilst less regular attenders talked more generally about helping and being helped as above. Regular attenders’ comments included life changing experiences and feelings, like those expressed in the following quotes:

The most powerful experience I’ve ever had in my life is that leadership program, you know, that changed my life, that changed my thinking … you know, make good decisions… they give you a good opportunity for you to be a good leader in the community (FS, Male).

*I: You’re officially coaching … how does that feel for you?R: It feels amazing. I look after people, then the people listen to me in the game and stuff (FS, Male).*

There was some mention of prosocial behaviour in the interviews at Comparison schools, in response to more direct questions about how interviewees would help other new arrivals to the IEC school. The matrix query showed eight Comparison Group interviewees talked about being helpful, in general terms with only three students, who were student leaders in their schools, mentioning concrete examples of the types of prosocial behaviours measured in the survey. For example

To help them, when they arrived, I actually a student leader. So my job is to help the students, so when the other students come, I’m trying to be friendly for them (CS, Female).

#### Resilience

The resilience scale showed no difference between young people in the FUn and Comparison Group. In the qualitative interviews, participants were not specifically questioned about their response to change or adversity, and no experiences or feelings which showed ‘resilience’ in the way it was measured in the chosen scale were found in the data set for either Program or Comparison school interviewees.

## Discussion

Young people in the FUn Group overall, and participating boys in particular, showed significantly more positive levels of other-group orientation than the Comparison Group, and some evidence (though lower in power and medium effects only) that the program positively impacted on peer problems and prosocial behaviour for boys. These quantitative results are well supported by the qualitative data from both groups and together the findings provide strong support for the effects of Football United on these key domains of peer and prosocial relationships for boys, and other-group orientation for young people in the program. The findings are of international significance as they show the positive impact of a sport-for-development program on important peer and social domains among young people using multiple triangulated methods [27].

We did not find impacts on resilience, the reporting of emotional symptoms or hyperactivity. These non-significant findings are not illuminated by the interview data in which young people did not discuss these constructs in any detail. With a maximum score of five for resilience, both the Program and Comparison group samples have a high mean score on this scale, which may mean most of these young people already had a high degree of resilience given their experiences prior to arriving in Australia making this variable less open to change. In terms of emotional symptoms, we did find that girls reported a significantly higher score on emotional symptoms than boys, and the impact of the program on girls, including on emotional symptoms, is an area for further investigation given the small number of girls recruited for the present study.

Treatment partitioning analyses indicated clear, positive, linear associations between other-group orientation and level of total participation in the Football United program, whilst reporting of peer problems and prosocial behaviour was associated with level of regularity of attendance. This is inherently logical if one considers attending regularly would be associated with fewer peer problems and better prosocial behaviour, but changing a person’s other-group orientation would require a large enough “dose” of positive exposure to other groups. Further, the greater the level of participation and regularity of attendance in Football United activities, the better participating young people reported feeling since commencing with the program. These linear associations suggest that the observed impacts are the result of the level of participation and attendance in the program, and not just the young people’s attendance at a Football United program school. It appears that the Football United program provides opportunities for many young people with a prosocial disposition to turn that disposition into actual behaviour – opportunities not generally available for the Comparison Group, except through things like school leadership programs which would only be available to a few newly arrived young people.

In the published literature we found little robust evidence of the effects of participation in specific sport-for-development programs on the participants themselves, which echoes other researchers’ conclusions about the paucity of evaluation studies [16]. We found many claims for positive impacts of local sport in more general terms, including on individual self-confidence and community cohesion from literature reviews, surveys of local authority staff and community residents [7,43]. However, reviews of past program evaluation studies have concluded that most evaluations rest on rigid matrices and rely on quantitative output data which does not capture the potential impacts on interpersonal relationships and skills [16]. The weaknesses of past evaluation efforts underscore the importance of our findings on peer and prosocial behaviour as key variables that show promise in evaluating the effects of sport-for-development programs.

### Peer relationships

Evaluation studies of sport-for-development programs focus almost exclusively on concepts such as social capital and often pay little attention to the effects on participants’ relationships with each other [16,44]. In the sport literature more generally, we only found one exploratory study in Canada that specifically looked at peer and social relationships and how they may be fostered through organised sport (soccer) [45]. This Canadian study found learning to interact with different types of peers and manage conflict could result from participation, although the sport setting studied was not specifically designed to maximise these effects [45].

### Prosocial behaviour

Studies of sport-for-development programs generally do not focus on evaluating prosocial behaviour as a specific impact, though programs may aim to foster these behaviours [16,44]. There have been mixed results from studies in the general sport literature that have examined how sport may influence prosocial and antisocial behaviour [45,46]. One relevant recent study looked at prosocial behaviour in adolescent soccer players involved in organised youth (boys) soccer in Amsterdam [46]. The study used validated measures of relational support and on and off-field antisocial and prosocial behaviour (using a different, but related measure to the prosocial measure used in the Football United study) and found prosocial behaviour was influenced by relational support provided by the coach and the team attitude towards fair play [46]. Other studies support the role of coaches in promoting pro-scoial behaviour [42]. In our study, we cannot specifically point to the role of coaches as the main factor in our findings on prosocial behaviour, though many interviewees commented on the satisfying experience they had as coaches and of being coached. How coaches’ roles are defined, supported and developed in programs like Football United is an important area of future investigation, including their impact on prosocial behaviour.

### Other-group orientation

Other-group orientation has been much less studied as a concept than ethnic identity in adolescent and migrant studies in general [47]. We found no studies in the published literature that looked at the effects of a sport-for-development program on a validated measure of other-group orientation, beyond more generalised claims for impacts on community cohesion [7] highlighting the unique finding about this construct from the current study. Examining how other-group orientation can be fostered through programs like Football United is an area for further exploration in our data set.

### School and community level impacts

The effects of Football United on the school environment in general, beyond just for those who directly participated in the program, is supported by some of our findings. In particular, a lack of peer problems was reported by some interviewees who were enrolled, but did not participate in any Football United activities. But there was also evidence that bullying was a problem at all schools. There is a need for further research to look at broader school level impacts of program such as Football United as school is a critical social field for young people in general and for early settlement [48].

There was no evidence of impact on the general measures of neighbourhood social inclusion used in our study. The data from the FUn Group revealed that the students lived in a number of neighbourhoods, some very geographically distant from the IEC school they attended. The finding that there was no difference on social inclusion variables, which were focussed on the neighbourhood level, is therefore not surprising. The social inclusion variables may therefore be considered to better represent each young person’s broader experiences rather than a possible outcome of the program. These measures may still be useful in evaluating other programs if most of the young people in a sport-for-development program reside in close proximity to each other [15].

### Experience of girls

Gender significantly impacted on reporting of emotional symptoms overall, with girls reporting a higher score than boys. Being more anxious and unhappy (the types of emotions we measured) may impact on girl’s engagement with Football United. Cultural barriers regarding girls’ engagement in sport in general, and with boys specifically, were noted as reasons for minimal female participation at the study sites and are prominent issues raised in the literature [49]. However, the qualitative data presented in this paper suggest at least some of the girls who participated in Football United enjoyed their experience. Other literature shows the potential for team sport to develop self-esteem among adolescent girls [50] providing further impetus for efforts to engage girls in future program design and to evaluate these efforts more fully.

#### Limitations

There were some study limitations and methodological changes made during the study conduct that are important to note. The power of the quantitative analysis of impacts was limited by the number of participants. The number of participants was limited to the number of young people participating in the Football United program at the sites chosen in the main study year - 2011. Further, study outcome data were only collected at one point in time, and program participation data for the one study year. Although treatment partitioning analyses were undertaken to assist in attributing impacts to participation in the Football United program, the time period for the study meant that it was not possible to determine whether other impacts (positive or negative) may result from longer participation in the Football United program, nor whether the impacts achieved would be sustained. As noted in the literature, many programs depend on “insecure and time-limited resources” (p133) [15]. Football United is no exception, needing to continually seek longer-term secure funding. Nonetheless, undertaking both an intention to treat and treatment partitioning analyses did allow consideration of three potential sources of program impact: being in a Football United school; the total amount of participation in the Football United program; and the average, regularity or attendance at Football United activities. In terms of sample composition it is also important to note that the Comparison group was similar, but not directly matched to the Program group though differences in demographic and immigrant experiences between the two groups had no impact on the significant findings reported in the paper.

The composite survey instrument was developed with significant attention to previous use of included instruments in Australia and among different language and cultural groups (see Additional file 1). Available psychometric studies (including internal and external construct validty and reliability, including inter-rater agreement and test/re-test) [32,33,35,37] were reviewed and face validity and pilot testing undertaken in the current study with key informants and young people at participating schools. The previously reported psychometric properties of the instruments were however limited to certain language groups. Face validity, pilot testing and analysis/comparison of qualitative data from a sub-sample of the young people who completed the survey increased confidence among the team that the survey findings were a valid reflection of the actual experiences of the young people in the study.

In the interviews, social desirability, which may lead young people to be more positive in their comments about schools (for both groups) and about the Football United program (for FUn Group only) could have been a factor. Specific techniques were used in interviews to lessen this effect and the finding that many qualitative comments supported the significant effects found using validated and reliable survey instruments gives more confidence that the interview comments reflected young people’s views and experiences not simply a desire to be liked and to say what may have been perceived as desirable. The triangulated methods also gives support to the accuracy of the assisted interpretations by the bi-lingual workers which occurred at some points during the qualitative interviews with a number of participants and was provided for some participants in completing the survey. The trust and rapport between bi-lingual staff and the participants (the staff work with these students daily to support them in learning English), was seen as a strength of the interview process. Trust and rapport with support staff would likely have enhanced the communication of young people’s experiences, moreso than would have been achieved with external translators who would not have an ongoing relationship with, or understanding of the experiences of the participants. A second interview was also originally proposed with the Football United students to examine experience of the program over time. However, this second interview was not undertaken for two key reasons. Firstly, the difficulty in arranging consent and scheduling a single interview which impacted on school staff time more than expected. Secondly, the data from the first interviews, which included students with a range of times of involvement in Football United, was judged by the team to have provided sufficient insight into young people with differing levels of exposure and time in the program.

## Conclusions

We agree with other scholars that no one program can address the broader macro structural factors which exclude individuals and communities from equal opportunity in sport and other life endeavours [15,48]. However, the study findings have contributed to the evidence base for the effectiveness of a holistic, integrated sport-for-development program in promoting cross-cultural relationships, and building peer and prosocial relationships and behaviours, particularly for young men. Fostering peer interactions through sport is undoubtedly important as acceptance by peers among adolescents from migrant backgrounds can be a precursor to feelings of self-worth and can improve overall happiness [51,52].

The impacts on reported peer relationships, prosocial behaviour and other-group orientation from the Football United study are of international significance and point to the need for other sport-for-development evaluation studies to include these variables in their measures. Further analysis is also needed to examine which program elements contribute most to these effects and how elements, such as specific training and mentoring, may foster these behaviours and attitudes. A specific focus on engaging girls in sport-for-development programs and evaluating these efforts is recommended. Much has also been learned through the research about appropriate approaches to evaluation that can help researchers capture the complexity of sport-for-development programs.

## Competing interests

ABB and TS were involved in program delivery in the study year. The other authors declare that they have no competing interests.

## Authors’ contributions

SN, LK, ABB, CE and JM all participated in the study conceptualisation and refinement of the design and measures. JM, CE and SN were involved in data collection. ABB and TS were involved in program delivery in the study year. LK analysed quantitative data with support from TS. JM undertook initial qualitative analysis with support from SN. All authors participated in the refinement of the qualitative data analysis and presentation in the paper. SN, LK , JM and ABB drafted the paper with SN leading the drafting and revision process. All authors have read, commented and approved the final manuscript.

## Pre-publication history

The pre-publication history for this paper can be accessed here:

http://www.biomedcentral.com/1471-2458/13/399/prepub

## Supplementary Material

Additional file 1 Instruments and items chosen for survey and rationale for choosing. Click here for file
