# Supplementary material for: “We wouldn’t of made friends if we didn’t come to Football United”: the impacts of a football program on young people’s peer, prosocial and cross-cultural relationships
Source: BMC Public Health. 2013 Apr 27;13:399. doi: 10.1186/1471-2458-13-399 (PMC3649946; doi:10.1186/1471-2458-13-399)
Supplement: Additional file 1 — Instruments and items chosen for survey and rationale for choosing. [file 1471-2458-13-399-S1.docx]

**Instruments and items chosen for survey and rationale for choosing**

| **Instrument** | **Outcome measured** | **Items** | **Rationale for choosing** | |  |
| --- | --- | --- | --- | --- | --- |
| Strengths and difficulties questionnaire – SDQ [1] | Emotional symptoms,  conduct problems (removed),  hyperactivity/inattention,  peer relationship problems  pro-social behaviour and  intervention impact (adapted from SDQ Impact supplement)  <http://www.sdqinfo.org/a0.html> | All items response categories: 1=not true, 2=somewhat true, 3=certainly true. Maximum score on each scale is 10. *Emotional symptoms* (higher score is more emotional symptoms on this scale): I get a lot of headaches, stomach-aches or sickness; I worry a lot; I am often unhappy, depressed or tearful; I have many fears, I am easily scared. I am nervous in new situations. I easily lose confidence. *Peer relationship problems* (lower score is less peer problems reported): I would rather be alone than with people of my age; I have one good friend or more; Other people my age generally like me; I get along better with adults than with people my own age; Other children or young people pick on me or bully me. *Pro-social* (higher score is more pro-social behaviours reported): I try to be nice to other people. I care about their feelings; I usually share with others, for example CD’s, games, food; I am helpful if someone is hurt, upset or feeling ill; I am kind to younger children; I often volunteer to help others (parents, teachers, children). *Hyperactivity* (higher score is more hyperactivity symptoms reported): I am restless, I cannot stay still for long; I am constantly fidgeting or squirming; I am easily distracted, I find it difficult to concentrate; I think before I do things; I finish the work I'm doing. My attention is good. | - The SDQ is widely used as a research tool internationally in developmental, social, and educational studies. - A wide range of published psychometric data available including internal and external construct validity and reliability - including inter-rater agreement and test/re-test [2]. - Translated and used in over 50 languages. - Australian comparative data available [3]. - Language simple and clear compared to other related scales. - Each of the sub-scales includes 5 items allowing separate scores to be calculated. - Use is free. | |  |
| Connor-Davidson Resilience Scale (CD- RISC) | Resilience | Two items about adaption to change and coping with life’s hardships with response categories from 1 to 5 with 1=not true at all; 2=rarely true; 3= sometimes true; 4= often true; 5= true nearly all of the time (higher score is more resilient on this measure). | - Published psychometric data available including reliability, validity, and factor analytic structure [4, 5] . - A short 2 item version CD-RISC2 available helping to reduce composite instrument length with internal consistency, test-retest reliability, convergent and divergent validity as well as significant correlation with the full scale demonstrated [5]. - Small usage fee | |  |
| Multigroup Ethnic Identity Measure and Other Group Orientation Scale | Ethnic identity (removed)  Other-group Orientation | 6 other-group orientation items with responses from 1 = strongly agree to 5 = strongly disagree, such as “I like meeting and getting to know people from ethnic groups other than my own” and “I sometimes feel it would be better if different ethnic groups didn't try to mix together” (lower score means a greater level of other-group orientation). | - Published psychometric data available [6-9]. - Used in the Good Starts Study – Victoria, Australia [10, 11]. - Use is free | |  |
| Selected items from the Health & Participation Survey [12] | Feelings of social inclusion | Two items with yes/no response: Do you have family members who live in your neighbourhood, but not in your house?; Do you have close friends who live in your neighbourhood? Third item asked: Which of the following fits your situation? 4 response options including; I know most of the people living in my neighbourhood; I know many of the people living in my neighbourhood; I know a few people in my neighbourhood, but most are strangers; I do not know people in my neighbourhood. | | - 3 items (items 12,13 &15) chosen that examine connections in neighbourhood with family and friends. - Designed for adult population so questions chosen were most appropriate for young people. - No national data, but smaller geographic areas available. - Psychometric data not available, as instrument does not measure a personality or mental health construct. - Use is free. | |
| Selected items from NSW Child Health Survey [13] | Feelings of social inclusion | Most people can be trusted. Do you...Four response options from strongly agree to strongly disagree with lower score meaning higher levels of trust in response to this question.  When you go out in your local area how often are you likely to run into friends?  Four response options from nearly always to rarely or never with lower score meaning report more likely to run into friends. | | - Designed for parents/carers of children - 2 items chosen (Items 246, 250) which were modified for young people. - Has been translated and used in other languages. - Psychometric data not available, as instrument does not measure a personality or mental health construct. - Use is free. | |

**References:**

1. Strengths and Difficulties Questionnaire [http://www.sdq.info]

2. Goodman R: **Psychometric Properties of the Strengths and Difficulties Questionnaire**. *Journal of the American Academy of Child & Adolescent Psychiatry* 2001, **40**(11):1337-1345.

3. Mellor D: **Normative data for the strengths and difficulties questionnaire in Australia**. *Australian Psychologist* 2005, **40**(3):215 - 222.

4. Connor KM, Davidson JRT: **Development of a new resilience scale: The Connor-Davidson Resilience Scale (CD-RISC)**. *Depression and Anxiety* 2003, **18**(2):76-82.

5. Vaishnavi S, Connor K, Davidson JRT: **An abbreviated version of the Connor-Davidson Resilience Scale (CD-RISC), the CD-RISC2: Psychometric properties and applications in psychopharmacological trials**. *Psychiatry Research* 2007, **152**(2-3):293-297.

6. Phinney JS: **The Multigroup Ethnic Identity Measure: A New Scale for Use With Diverse Groups**. *Journal of Adolescent Research* 1992, **7**(2):156-176.

7. Phinney JS, Jacoby B, Silva C: **Positive intergroup attitudes: The role of ethnic identity**. *International Journal of Behavioral Development* 2007, **31**(5):478-490.

8. Phinney JS, Ong AD: **Conceptualization and Measurement of Ethnic Identity: Current Status and Future Directions**. *Journal of Counseling Psychology* 2007, **54**(3):271-281.

9. Ponterotto JG, Gretchen D, Utsey SO, Stracuzzi T, Saya Jr. R: **The Multigroup Ethnic Identity Measure (MEIM): Psychometric Review and Further Validity Testing**. *Educational and Psychological Measurement* 2003, **63**(3):502-515.

10. **Settlement Journal: Good Starts Study for Refugee Youth**. In*.* La Trobe University: Refugee Health Research Centre, Australia; 2004.

11. Gifford S, M., Bakopanos C, Kaplan I, Correa-Velez I: **Meaning or Measurement? Researching the Social Contexts of Health and Settlement among Newly-arrived Refugee Youth in Melbourne, Australia**. *Journal of Refugee Studies* 2007, **20**(3):414.

12. Baum F, Bush R, al E: **Building Healthy Communities: Health Development and Social Capital Project**. In*.*: SA Community Health Research Unit and Department of Public Health, Flinders University of SA, Australia.; 1999.

13. Centre for Epidemiology and Research: **New South Wales Child Health Survey 2001**. *NSW Public Health Bulletin* 2002, **13**(3):1-84.
